# Dental fear, tobacco use and alcohol use among university students in Finland: a national survey

**DOI:** 10.1186/1472-6831-14-86

**Published:** 2014-07-11

**Authors:** Vesa Pohjola, Lauri Rannanautio, Kristina Kunttu, Jorma I Virtanen

**Affiliations:** 1 Department of Community Dentistry, Institute of Dentistry, University of Oulu, Oulu, Finland; 2 Finnish Student Health Service, Turku, Finland; 3 Oral and Maxillo-Facial Department, Oulu University Hospital, Oulu, Finland

**Keywords:** Alcohol use, AUDIT, Dental fear, Students, Tobacco use

## Abstract

**Background:**

Tobacco- and alcohol use are associated with psychological problems. Individuals with high dental fear also more often report other psychological problems than do those with lower level of dental fear. We evaluated the association between dental fear and tobacco- and alcohol use while controlling for age, gender, general mood and feelings in social situations.

**Methods:**

The data (n = 8514) were collected from all universities in Finland with an electronic inquiry sent to all first-year university students. Dental fear was measured with the question: “How afraid are you of visiting a dentist?” with reply alternatives “Not at all”, “Somewhat” and “Very”. Regularity of tobacco use was determined with the question: “Do you smoke or use snuff?”, with reply alternatives “Not at all”, “Occasionally” and “Daily”. The Alcohol Use Disorders Identification Test (AUDIT) was used for determination of alcohol use; an AUDIT sum score of 8 or more indicated hazardous, harmful or dependent alcohol use. The statistical tests used were Chi-square tests and Multiple logistic regression analyses.

**Results:**

When controlled for age, gender, alcohol use, general mood and feelings in social situations, those who used tobacco regularly were more likely to have high dental fear than were those who used tobacco occasionally or not at all. When controlled for age, gender, general mood and feelings in social situations, those with hazardous, harmful or dependent alcohol use were more likely to have high dental fear than were those with low-risk of alcohol use, the association between alcohol use and dental fear was not strong. When tobacco use was added into this model, alcohol use was no longer statistically significantly associated with dental fear.

**Conclusions:**

The findings of this study support the suggestion that some people may have common vulnerability factors linked to tobacco use, alcohol use, and dental fear.

## Background

Vulnerability to dental fear has been suggested to be related to other psychological problems in some individuals [1-5]. People with high dental fear more often report psychological problems, such as anxiety disorders, mood disorders and alcohol dependence, than do people with lower level of dental fear [1,4,5]; this has been found both in a young adult population [1] and in an adult population [4,5]. Cognitive vulnerability provides a way of understanding the factors that contribute to the onset, recurrence and maintenance of psychological problems [2,3]. Vulnerability factors have been reported to be risk indicators for alcohol dependence [6], and nicotine dependence has also been associated with vulnerability to psychopathology [7].

Problems related to alcohol use and anxiety often occur in the same individuals [8-12]. Both in the general population [11] and among students [12] the rate of anxiety has been reported to be higher in individuals with alcohol use disorders. Among students who drink moderately, anxiety is more often been reported by males than by females [13]. In addition, people with alcohol dependence were more likely to have high dental fear than were those without alcohol dependence [5].

Similar to alcohol use, use of tobacco is associated with psychological problems [14,15]. Depression is more often been reported by students who smoke regularly than by non-smoking students [16-18]. Smokers more commonly report anxiety than non-smokers do [15,19]; this has also been found among students [17]. High dental fear is more often been reported by people who smoke regularly than by those who smoke occasionally or not at all [5].

The association between alcohol use and tobacco use and dental fear may be affected by age, gender and socio-economic status. A finding that is highly consistent across studies is that the rates of alcohol use disorders among men are nearly double those of women [20,21]. In addition, studies have shown a general pattern of higher smoking prevalence among groups with low socio-economic status [22]. Younger age groups, women and individuals with low educational level more commonly report dental fear than older age-groups, men and individuals with high educational level do [23-25]. Educational level has been seen as one indicator of socio-economic status [22]. Young students starting their first year at university are also in transition to adulthood. This period of life involves an extended period of learning and experimentation, which may affect health behaviors [26], e.g. alcohol use and smoking.

Dental fear, tobacco use and alcohol use are all associated with other psychological problems, but there have been few studies of the association between dental fear, tobacco use and alcohol use [1,5]; and as far as we know, this has not been studied in a student population. Differences between age - and socio-economic groups in terms of dental fear [23-25], alcohol use [20,21] and smoking [9,22] are known, and a study among students in Finland might give different results compared to one among adults aged 30 years and older [5]. Thus, the aim of this study was to evaluate the association between dental fear, tobacco use and alcohol use among students in Finland. Our hypotheses were that tobacco use and alcohol use are associated with dental fear and that high dental fear is more common among regular tobacco users than among occasional- or non-tobacco users and among those with hazardous, harmful or alcohol-dependent consumption of alcohol than among those with low-risk for alcohol use.

## Methods

This cross-sectional study was carried out among Finnish university students, who are entitled to subsidized health care services, including dental care, provided by the Finnish Student Health Service (FSHS). All first-year university students are sent an electronic health questionnaire inquiring about their health and health habits. This questionnaire was e-mailed in Finnish, Swedish (Finnish and Swedish are official languages in Finland) or English (less than 4% of the university students in Finland are international students). Each student receives a personal answer to the electronic health questionnaire. If the inquiry shows problems in the student’s health or health habits, he/she is invited to a health examination. The data for this study were collected from electronic health inquiries answered by university students who started their studies in autumn 2011. Permission for the study was given by the authorities of FSHS and approval was given by the Ethics Committee of the Northern Ostrobothnia Hospital District. All universities in Finland were included in the study. In 2011 there were 17 091 first-year students in Finland; and the electronic health inquiry was answered by 8514, i.e. 50%, of the first-year students.

Dental fear was covered by a single question: “How afraid are you of visiting a dentist?”. The reply alternatives were “Not at all”, “Somewhat” and “Very”. Later the alternatives ‘Not at all’ and ‘Somewhat’ were combined into a category indicating low or no fear and ‘Very’ was used as the category for high level of fear. This categorization was because high dental fear has the most severe clinical consequences for dental attendance and dental health. Measuring dental fear with this single question has been shown to be valid and reliable [27]. Regularity of smoking or use of snuff was determined by the question: “Do you smoke or use snuff?”, with three reply alternatives: “Not at all”, “Occasionally” and “Daily”. In this study snuff referred to all forms of smokeless tobacco. The Alcohol Use Disorders Identification Test (AUDIT), was answered by those students who answered “Yes” to the question: “Do you use alcohol?”. AUDIT is a 10-item questionnaire that covers alcohol consumption, drinking behaviour and alcohol-related problems. In this study an AUDIT score of 8 or more indicated hazardous, harmful or alcohol-dependent use of alcohol. AUDIT has been shown to be a valid and reliable measurement of alcohol use [28-30].

For the analyses, age was categorized into six groups: 19 or younger, 20, 21–24, 25–29, 30–39 and 40+ years. The youngest age group has entered the university directly from high school, the second age group one year after high school (boys often after military service) and the other age groups may have been working or studying something else before entering the university. General mood was determined with the question: “How is your mood in general?”. The reply alternatives were on the scale −10 to +10, where answers 1 to +10 indicated “Positive mood”, 0 “Neutral mood” and −10 to −1 “Negative mood”. Feelings in social situations was determined by the question: “How do you feel in social situations (e.g. giving an oral presentation)?”. The reply alternatives were on the scale −10 to +10 and answers 1 to +10 indicated “Positive feelings”, 0 “Neutral feelings” and answers −10 to 0 “Negative feelings”. The questions about feelings in social situations and general mood have been used in many studies in FSHS since 1979 [21].

### Statistical analysis

Bivariate associations between dental fear and age, gender, general mood and feelings in social situations, tobacco use and alcohol use were evaluated. The statistical significances of differences in the bivariate associations were evaluated with Chi-square tests.

Multiple logistic regression analyses were used to evaluate the association between dental fear and alcohol use disorders, controlling first for the effect of age and gender. Then the analyses were repeated, adding general mood and feelings in social situations and finally, adding tobacco use to the model. The analyses were performed as in the previous studies [1,5]. Age was entered into the multiple logistic regression analysis as a continuous variable. The level of statistical significance was set at p < 0.05. The analysis was performed with SPSS, version 20.0 [31].

## Results

Among Finnish university students, 5.4% reported high dental fear, which was more common among women (6.9%) than among men (2.5%) (Table 1). Negative general mood was reported by one out of twenty and negative feelings in social situations by two out of ten students. Regular tobacco use was more common among men (9.5%) than among women (4.6%). The prevalence of alcohol use was similar in men (80.7%) and women (79.0%), but an AUDIT sum score of 8 or higher was more common among men (47.6%) than among women (25.7%).

**Table 1:** Description of the sample of Finnish university students (n = 8514)

	**n**	**All %**	**Men %**	**Women %**
**Gender**				
Men	2958	34.7		
Women	5556	65.3		
**Age**				
≤19	2516	29.6	19.9	34.7
20	2150	25.3	30.3	22.6
21–24	2164	25.4	30.1	22.9
25–29	927	10.9	12.4	10.1
30–39	481	5.6	5.2	5.9
40+	276	3.2	2.1	3.9
**Dental fear**				
Very afraid	459	5.4	2.5	6.9
Somewhat afraid	2836	33.3	25.0	37.7
Not at all afraid	5219	61.3	72.5	55.3
**How is your general mood?**				
Positive (1 to +10)	7641	89.7	89.5	89.9
Neutral (0)	310	3.6	4.3	3.3
Negative (−10 to −1)	563	6.6	6.2	6.8
**How do you feel in social situations (e.g. giving a presentation)?**				
Positive (1 to +10)	6133	72.1	74.2	70.9
Neutral (0)	617	7.2	7.9	6.9
Negative (−10 to −1)	1769	20.7	17.9	22.2
**Do you smoke or use snuff?**				
Regularly	539	6.3	9.5	4.6
Occasionally	1275	15.0	17.6	13.6
Not at all	6700	78.7	72.9	81.8
**Do you use alcohol?**				
Yes	6777	79.6	80.7	79.0
No	1737	20.4	19.3	21.0
**Alcohol AUDIT**^**1 **^**(sum score)**				
7 or less	4514	66.6	52.4	74.3
8 or more	2263	33.4	47.6	25.7

^1^Alcohol AUDIT questionnaire was answered by those participants who answered “Yes” to the question “Do you use alcohol?” (n = 6777).

Younger students (4.0%) reported high dental fear less often than older students did (6.9%, p < 0.001) (Table 2). Students who reported negative mood (6.9%) or negative feelings in social situations (8.0%) also reported high dental fear more often than those who reported positive mood (5.1%, p = 0.001) or positive feelings in social situations (5.1%, p < 0.001). Regular smokers (9.5%) were more often very afraid of visiting a dentist than were those who smoked occasionally (5.8%) or not at all (5.0%, p < 0.001). Among men, those who did not use alcohol (3.5%) reported high dental fear more often than who used alcohol (2.5%, p = 0.029). The prevalence of high dental fear was similar among those whose AUDIT sum score was 8 or more (5.5%) compared to those with AUDIT sum score of 7 or less (5.3%).

**Table 2:** Prevalence of university students according to level of dental fear (n = 8514)

	**Dental fear among all**				**Dental fear among women**				**Dental fear among men**			
	**Very afraid**	**Some-what afraid**	**Not at all afraid**	***p***	**Very afraid**	**Some-what afraid**	**Not at all afraid**	***p***	**Very afraid**	**Some-what afraid**	**Not at all afraid**	***p***
**Age**	%	%	%		%	%	%		%	%	%	
	(n)	(n)	(n)		(n)	(n)	(n)		(n)	(n)	(n)	
≤19	4.0	31.8	64.2	<0.001	4.7	34.7	60.6	<0.001	1.5	22.3	76.2	<0.001
	(100)	(800)	(1616)		(91)	(669)	(1168)		(9)	(131)	(448)	
20	4.4	30.6	65		6.2	37.8	55.9		1.9	20.4	77.7	
	(95)	(657)	(1398)		(78)	(474)	(701)		(17)	(183)	(697)	
21–24	6.8	34.8	58.4		9.8	40.2	50		2.6	27.1	70.3	
	(148)	(753)	(1263)		(125)	(512)	(638)		(23)	(241)	(625)	
25–29	6.5	37.4	56.1		7.7	41.4	50.9		4.6	31.3	64	
	(60)	(347)	(520)		(43)	(232)	(285)		(17)	(115)	(235)	
30–39	7.7	37.6	54.7		8.9	39.3	51.8		5.2	34.2	60.6	
	(37)	(181)	(263)		(29)	(128)	(169)		(8)	(53)	(94)	
40+	6.9	35.5	57.6		8.9	37.9	53.3		0	27.4	72.6	
	(19)	(48)	(159)		(19)	(81)	(114)		(0)	(17)	(45)	
**General mood**												
Positive	5.1	33.0	61.9	0.001	6.6	37.7	55.6	0.030	2.3	24.0	73.6	0.001
1 to + 10	(393)	(2521)	(4727)		(331)	(1884)	(2777)		(62)	(637)	(1950)	
Neutral	6.8	40.0	58.1		8.2	42.9	48.9		4.8	35.7	59.5	
0	(21)	(124)	(165)		(15)	(79)	(90)		(6)	(45)	(75)	
Negative	8.0	33.9	53.2		10.3	35.0	54.7		3.3	31.7	65.0	
−10 to −1	(45)	(191)	(327)		(39)	(133)	(208)		(6)	(58)	(119)	
**Feelings in social situations**												
Positive	5.1	31.9	63.1	<0.001	6.7	36.6	56.7	0.005	2.1	23.5	74.4	0.001
1 to + 10	(311)	(1955)	(3867)		(264)	(1140)	(2235)		(47)	(515)	(1632)	
Neutral	4.2	35.3	60.5		5.2	42.4	52.4		2.6	23.8	73.6	
0	(26)	(218)	(373)		(20)	(162)	(200)		(6)	(56)	(173)	
Negative	6.9	37.6	55.5		8.2	40.0	51.8		4.0	31.9	64.1	
−10 to −1	(122)	(663)	(979)		(101)	(494)	(640)		(21)	(169)	(339)	
**Tobacco use**												
Not at all	5.0	33.3	61.7	<0.001	6.4	37.2	56.4	<0.001	2.1	25	72.9	0.002
	(334)	(2231)	(4135)		(289)	(1692)	(2564)		(45)	(539)	(1571)	
Occasionally	5.8	33.3	60.9		8.1	40.3	51.6		2.5	23.2	74.3	
	(74)	(425)	(776)		(61)	(304)	(389)		(13)	(121)	(387)	
Regularly	9.5	33.4	57.1		13.6	38.9	47.5		5.7	28.3	66.0	
	(51)	(180)	(308)		(35)	(100)	(122)		(16)	(80)	(186)	
**Use of alcohol**												
No	5.4	35.1	59.6	0.217	6.3	38.5	55.3	0.551	3.5	28.1	68.4	0.029
	(93)	(609)	(1035)		(73)	(449)	(645)		(20)	(160)	(390)	
Yes	5.4	32.9	61.7		7.1	37.5	55.4		2.3	24.3	73.5	
	(366)	(2227)	(4184)		(312)	(1647)	(2430)		(54)	(580)	(1754)	
**Alcohol AUDIT** (sum score)												
7 or less	5.3	33.8	60.9	0.083	6.7	37.4	56	0.093	1.9	24.4	73.7	0.497
	(241)	(1524)	(2749)		(217)	(1219)	(1827)		(24)	(305)	(922)	
8 or more	5.5	31.1	63.4		8.4	38.0	53.6		2.6	24.2	73.2	
	(125)	(703)	(1435)		(95)	(428)	(603)		(30)	(275)	(832)	

In the logistic regression analyses, when age and gender were controlled for, those with an AUDIT sum score of 8 or more were more likely to have high dental fear than were those with an AUDIT sum score of 7 or less (Table 3). In the corresponding gender-specific models, a similar association was found among women (Table 4). When feelings in social situations and general mood were also controlled for, those with an AUDIT sum score of 8 or more were more likely to have high dental fear than were those with an AUDIT sum score of 7 or less. In the corresponding gender-specific models, a similar association was also found among women (Table 4).

**Table 3:** Results of logistic regression analyses* (n = 6777)

	**Model 1**^**a**^			**Model 2**^**b**^			**Model 3**^**c**^		
	**OR**	**95% CI**	**p**	**OR**	**95% CI**	**p**	**OR**	**95% CI**	**p**
Alcohol use, AUDIT 7/8	1.3	1.1-1.7	0.011	1.3	1.04-1.6	0.024	1.2	0.9-1.5	0.151
General mood				1.5	1.1-2.2	0.034	1.4	1.0-2.1	0.066
Feelings in social situations				1.2	1.0-1.6	0.107	1.2	0.9-1.6	0.140
Tobacco use							2.1	1.5-3.0	<0.001

*The association between dental fear and alcohol use, general mood, feelings in social situations and tobacco use, reference groups = somewhat or not at all afraid, AUDIT sum score 7 or less, neutral or positive general mood, neutral or positive feelings in social situations, and using tobacco occasionally or not at all.

^a^Model 1 adjusted for age and gender. Nagelkerke r^2^ = 0.04.

^b^Model 2 adds General mood and Feelings in social situations. Nagelkerke r^2^ = 0.044.

^c^Model 3 adds Tobacco use. Nagelkerke r^2^ = 0.051.

**Table 4:** Gender-specific results of logistic regression analyses* (n = 6777)

	**Model 1**^**a**^			**Model 2**^**b**^			**Model 3**^**c**^		
	**OR**	**95% CI**	**p**	**OR**	**95% CI**	**p**	**OR**	**95% CI**	**p**
Men									
Alcohol use, AUDIT 7/8	1.4	0.8-2.4	0.217	1.4	0.8-2.4	0.249	1.2	0.7-2.1	0.545
General mood				1.1	0.4-3.2	0.874	1.0	0.3-2.9	0.962
Feelings in social situations				1.7	0.9-3.4	0.088	1.7	0.9-3.2	0.108
Tobacco use							2.8	1.5-5.3	0.002
Women									
Alcohol use, AUDIT 7/8	1.3	1.0-1.7	0.026	1.3	1.04-1.7	0.050	1.2	0.9-1.6	0.180
General mood				1.6	1.1-2.4	0.026	1.5	1.04-2.3	0.043
Feelings in social situations				1.2	0.9-1.5	0.286	1.1	0.9-1.5	0.334
Tobacco use							1.9	1.3-2.9	0.002

*The association between dental fear and alcohol use, general mood, feelings in social situations and tobacco use, reference groups = somewhat or not at all afraid, AUDIT sum score 7 or less, neutral or positive general mood, neutral or positive feelings in social situations, and using tobacco occasionally or not at all.

^a^Model 1 adjusted for age.

^b^Model 2 adds General mood and Feelings in social situations.

^c^Model 3 adds Tobacco use.

When, in addition to age and gender, alcohol use, feelings in social situations and general mood, tobacco use was added into the model, those who used tobacco regularly were more likely to have high dental fear than were those who used tobacco occasionally or not at all (Table 3). In this model, alcohol use was no longer statistically significantly associated with dental fear. This was found both in analyses that included all participants and when data were analyzed separately for men and women (Table 4). The association between dental fear and alcohol use was also studied separately among those who used tobacco occasionally or not at all and those who used tobacco regularly. The first models included age and gender, and the second models also included feelings in social situations and general mood as confounders (Table 5). In these models, among those who used tobacco occasionally or not at all, alcohol use was associated with dental fear; students with an AUDIT sum score of 8 or more were more likely to have high dental fear than were students with an AUDIT sum score of 7 or less.

**Table 5:** Tobacco use status-specific results of logistic regression analyses* (n = 6777)

	**Model 1**^**a**^			**Model 2**^**b**^		
	**OR**	**95% CI**	**p**	**OR**	**95% CI**	**p**
Using tobacco occasionally or not at all						
Alcohol use, AUDIT 7/8	1.3	1.04-1.7	0.024	1.3	1.04-1.7	0.041
General mood				1.7	1.1-2.6	0.009
Feelings in social situations				1.2	0.9-1.5	0.304
Using tobacco regularly						
Alcohol use, AUDIT 7/8	0.7	0.4-1.3	0.257	0.7	0.3-1.3	0.211
General mood				0.6	0.2-1.7	0.301
Feelings in social situations				1.7	0.9-3.3	0.136

*The association between dental fear and alcohol use, general mood and feelings in social situations, reference groups = somewhat or not at all afraid, AUDIT sum score 7 or less, neutral or positive general mood and neutral or positive feelings in social situations.

^a^Model 1 adjusted for age and gender.

^b^Model 2 adds General mood and Feelings in social situations.

General mood was associated with dental fear; those who reported negative mood were more likely to have high dental fear than were those who reported positive mood (Table 3). In gender-specific models this was found among women even after tobacco use was added to the model (Table 4). In models specific for tobacco use status, a similar association was also found among those who used tobacco occasionally or not at all (Table 5). According to logistic regression analyses, feelings in social situations was not associated with dental fear (Tables 3, 4 and 5).

## Discussion

When age, gender, alcohol use, general mood and feelings in social situations were controlled for, tobacco use was associated with dental fear. Those who used tobacco regularly were more likely to have high dental fear than were those who used tobacco occasionally or not at all. When controlling for age, gender, feelings in social situations and general mood, alcohol use was associated with dental fear. Among those who used tobacco occasionally or not at all, participants with current hazardous, harmful or alcohol-dependent alcohol use were more likely to have high dental fear than were participants with low-risk of alcohol use. As far as we know, this was the first study on the association between dental fear and alcohol use assessed with AUDIT and controlling for tobacco use.

Smoking has been linked to anxiety in general [14,15,17,19]. Thus, it was not surprising that in this study regular tobacco users were more likely to have high dental fear than were those who used tobacco occasionally or not at all. The mechanisms by which anxiety disorders increase smoking behavior could include a propensity for those with increased anxiety to start smoking, or the use of tobacco as an anxiolytic self-treatment [15]. Tobacco use may mitigate anxiety symptoms over the short-term. However, the use of tobacco and/or alcohol predisposes people to development of anxiety over time by, e.g. producing chronic withdrawal symptoms and possible precipitation of somatic or emotional symptoms that maintain anxiety [32]. Interestingly, among those who used tobacco regularly, alcohol use was not associated with dental fear. The high prevalence of tobacco use among people with hazardous, harmful or alcohol-dependent alcohol use [10,33] and dental fear [5] might affect the association between alcohol use and dental fear. As tobacco- and alcohol use [10,33] and tobacco use and dental fear are associated [5], tobacco use could mediate the effect of alcohol use disorder on dental fear. However, because of the cross-sectional design of this study, no clear causal interpretations can be made. To determine whether tobacco use mediates the effect of alcohol use disorder on dental fear or is a confounding factor in this association, longitudinal studies are needed.

Hazardous, harmful or alcohol dependent drinking was more common among men than among women, which has also been found in earlier studies among students [21,34]. In a study with young adults, when the effect of gender was controlled for, Locker *et al.*[1] found that those with high dental anxiety were more likely than the less anxious to have a diagnosis of alcohol- and cannabis dependence. Among adults in Finland, when socio-demographics and anxiety and depressive disorders were controlled for, those with high dental fear were more likely to have an alcohol use disorder than were those with lower level of dental fear [5]. The results of our study support these earlier findings.

The association between alcohol use and dental fear was not strong; and in gender-specific models, alcohol use was associated with dental fear among women but not among men. In general, dental fear was more common among women than among men. Of 100 university students only five reported high dental fear; this is lower than the prevalence of high dental fear among adults in Finland [24]. Previous studies have also shown that level of education affects prevalence of dental fear; individuals with high level of education less often report high dental fear than do those with lower level of education [23,24]. In addition, it has been reported previously that gender and socio-economic status modify and/or confound the prevalence of mental disorders, smoking and alcohol use [20,22]. Gender and socio-economic status should be taken account when the association between alcohol use, tobacco use and dental fear is studied.

In this study, four out of five students reported using alcohol, and the prevalence of alcohol use was similar among men and women. Previously it has been reported that the peak period for prevalence of alcohol use disorders occurs in late adolescence and early adulthood [23], which is in concordance with the high prevalence of alcohol use found in this study. However, the majority of those with hazardous, harmful or alcohol-dependent alcohol use did not have high dental fear. This is also in agreement with previous findings [1,5].

Among most individuals with dental fear, treatment or vicarious experiences [35] may be more important in the development of dental fear than psychopathology is [1]. It has been suggested that anxiety disorders and smoking behavior may have a shared vulnerability factor or group of factors that increase the likelihood of smoking and of developing anxiety disorders [15]. Some individuals with dental fear might share this vulnerability factor or group of factors. People with psychological problems may also have common patterns of behavior, like smoking or use of alcohol, which they may use as self-medication, but our current understanding on this point is inconclusive [36]. In addition, previous studies have shown that genetic factors play an important role in the pathogenesis of alcohol dependence [37], that nicotine dependence is substantially heritable [38] and that there may be a genetic component in dental fear [39]. In the development of alcohol use problems, smoking habit and dental fear, there may be common vulnerability factors. People with these factors might belong to a group with ‘constitutional vulnerability’ to dental fear [1,40].

When age, gender, alcohol use, tobacco use and feelings in social situations were controlled for, those reporting negative general mood were more likely to have high dental fear compared to those reporting neutral or positive mood. General mood was used as an indicator of mood disorders. The finding of this study is in agreement with the results of a previous study where high dental fear was more commonly reported by those with high level of depression than by those with low level of depression [3]. In this study, feelings in social situations was not associated with dental fear. The question concerning feelings in social situations gave as an example “giving an oral presentation”. This might have turned the focus on performance-based social situations and placed less emphasis on other social situations involving interaction, such as talking as part of a small group. Fear of these different social situations may have a different effect on social behavior [41]. Results in studies of the association between phobias related to social situations and dental fear are inconsistent [1,4,23].

In this study there are both strengths and limitations. Dental fear was measured with a single item that has been shown to be valid and reliable in Finnish and Norwegian adult populations [27,42]. The large sample was collected from all universities in Finland; however, it did not include the rest of the Finnish young adult population. The electronic health inquiry was answered by half of the university students, which can be considered good participation. According to a meta-analysis, the mean response rate in the web survey was 34% [43]. The questionnaire was completed on the internet, which may have excluded some persons from the study. On the internet it is easy not to answer questionnaires as the respondent does not have to answer anyone in person. On the other hand, an electronic questionnaire might result in a more valid estimation of dental fear than if it were asked, e.g. in connection with a clinical examination, in which those with high fear might not participate. The question about tobacco use combined smoking and use of snuff. This question has not been validated; however, the prevalence of tobacco use in this study and in the Student Health Survey 2012 [21] were almost identical. Alcohol use was determined by the AUDIT questionnaire, which is a reliable and valid screening instrument [28-30]. Unlike most other alcohol screening tests, the AUDIT questionnaire has been specifically designed to identify current hazardous or harmful alcohol consumption or dependence on alcohol [26]. In this study almost half of the male participants had score that indicated hazardous, harmful or alcohol-dependent drinking of alcohol. In this population the AUDIT cutoff point (8 or more) might have been too low to identify those with real drinking problems. Multiple-item measures, like AUDIT [28-30], may have higher scores for reliability and validity than do single questions, e.g. for measuring dental fear [27,42]. On the other hand, multiple-item measures take a longer time to answer, which might discourage some people from answering. In addition, because the study is cross-sectional, no causal interpretations can be made; and it is not possible to know whether alcohol- or tobacco use increases dental fear or vice versa.

## Conclusions

Our results support previous findings that those who use tobacco regularly or have problems related to alcohol use were more likely to have high dental fear than those who use tobacco occasionally or not at all or have low-risk of alcohol use problems. This supports the suggestion that some individuals may have common vulnerability factors involved in development of dental fear, alcohol use problems and tobacco use [1,2,5]. Dentists should take vulnerability to dental fear, tobacco - and alcohol use into account when treating fearful patients, as these patients are at risk for several health problems. For instance, a brief tobacco intervention performed by a dentist can support other anti-smoking activities [44]. The dentist should co-operate, e.g. with general practitioners and psychologists, to help people who use tobacco, have problems with alcohol use and have dental fear.

## Competing interests

The authors declare that they have no competing interests.

## Authors’ contributions

VP: designed the study, performed statistical analyses and wrote the manuscript, LR: performed statistical analyses and wrote the manuscript, KK: collected data and wrote the manuscript. JIV: designed the study and wrote the manuscript. All authors read and approved the final manuscript.

## Pre-publication history

The pre-publication history for this paper can be accessed here:

http://www.biomedcentral.com/1472-6831/14/86/prepub
